# Vertebral artery contribution to cerebral cortex perfusion in cattle after slaughter by ventral neck incision: a systematic review

**DOI:** 10.3389/fvets.2026.1760260

**Published:** 2026-02-25

**Authors:** Jacob R. Hascalovici, Paolo Pozzi, Kathleen Yvorchuk, Guy St-Jean, Stuart D. Rosen

**Affiliations:** 1 Albert Einstein College of Medicine, New York, NY, United States; 2 Hackensack Meridian Health Neuroscience Institute, Hackensack, NJ, United States; 3 Dipartimento di Scienze Veterinarie, Università degli Studi di Torino, Torino, Italy; 4 Veterinary Consultant, Montreal, QC, Canada; 5 National Heart and Lung Institute, Imperial College London, London, United Kingdom

**Keywords:** blood flow, brain, carotid arteries, consciousness, cortex, halal slaughter, shechita, vertebral arteries

## Abstract

**Background:**

Jewish shechita and Islamic halal are distinct yet similar forms of slaughter by exsanguination via ventral neck incision (SEVNI); neither permits preslaughter stunning. SEVNI has been criticized on the grounds that the vertebral arteries in cattle, which remain intact after SEVNI, may continue to supply blood to the brain, potentially delaying loss of consciousness (LOC) and causing unnecessary pain and distress to the animal. In this context, LOC is the loss of cortical awareness, which by definition abolishes sensibility and pain perception. The objective of this review is to evaluate the literature that specifically addresses the role of the vertebral artery in brain perfusion following SEVNI.

**Methods:**

This study was not funded. A non-registered systematic search of PubMed, Google Scholar, the Cochrane Library, Medline, and Web of Science (last searched 02/10/2026) identified experimental, observational and physiological studies that assessed vertebral artery hemodynamics and/or evaluated the functional significance of the vertebral artery in cattle. Non-cattle studies, studies lacking relevant measures, reviews, commentaries, and abstracts without full text were excluded. Quantitative pooling (meta-analysis) was not performed due to methodological and outcome variability therefore results were synthesized narratively.

**Results:**

Thirteen studies were included, with all articles independently reviewed by co-authors to minimize risk of bias. Using the ROBINS-I framework, the overall risk of bias across included studies was assessed as moderate. Across the reviewed studies, evidence consistently demonstrated that immediately following SEVNI, vertebral blood flow and pressure decrease to negligible levels, with most residual flow diverted away from the cerebral cortex.

**Limitations:**

Study limitations include heterogeneous study designs, variable outcomes and methods.

**Conclusion:**

The available evidence indicates that vertebral artery flow following SEVNI is unlikely to be sufficient to sustain cortical perfusion, support integrated cortical function or delay LOC.

## Introduction

**Figure 1:**
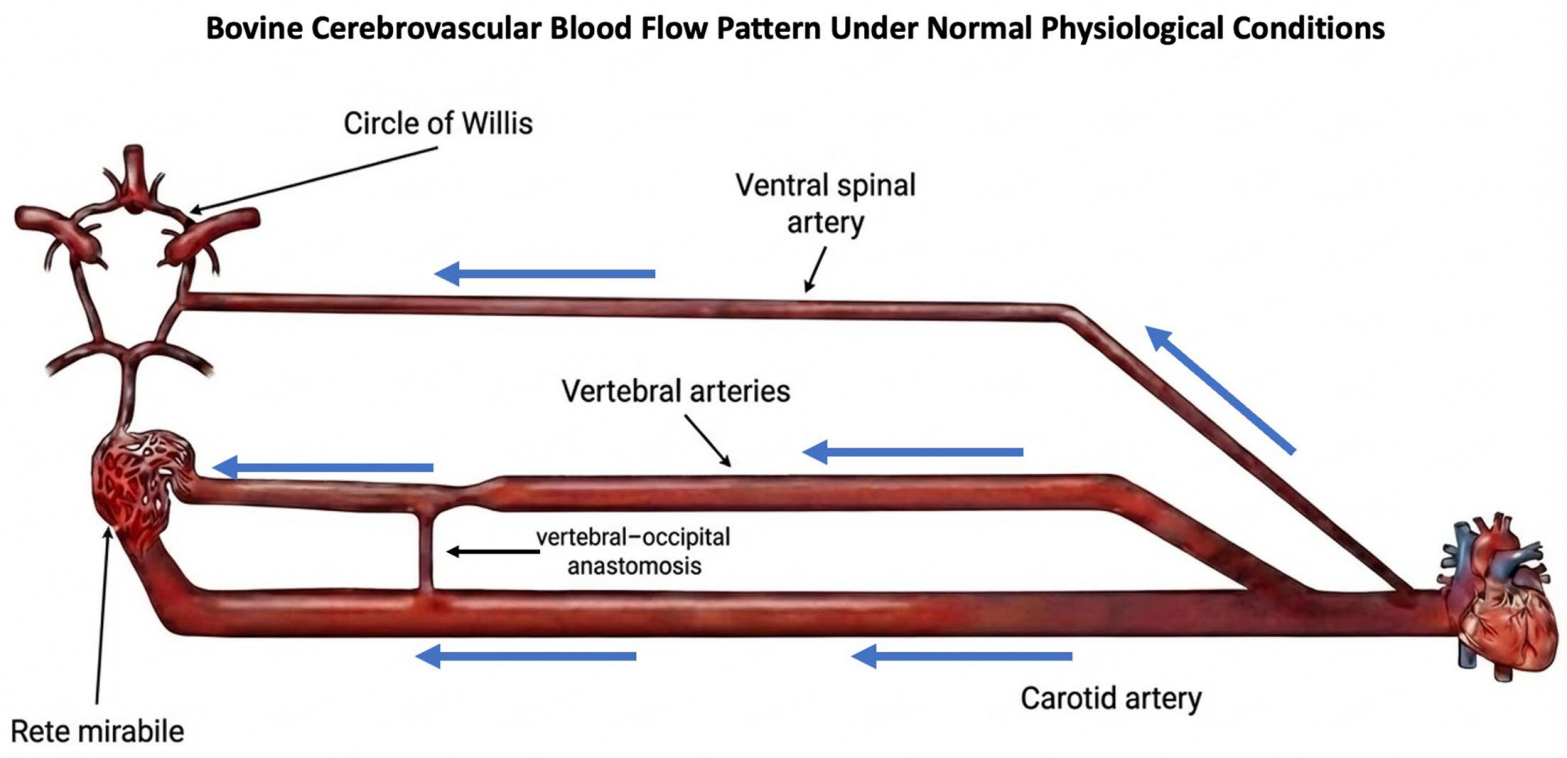
Depiction of the major arterial pathways supplying the bovine brain under intact physiological conditions. Cerebral perfusion is primarily driven by carotid arterial inflow to the rete mirabile, with contributions from the vertebral arteries via the ventral spinal artery and connections to the Circle of Willis. The illustration emphasizes anatomical relationships within a closed circulatory system. Blue arrows represent normal flow direction.

**Figure 2:**
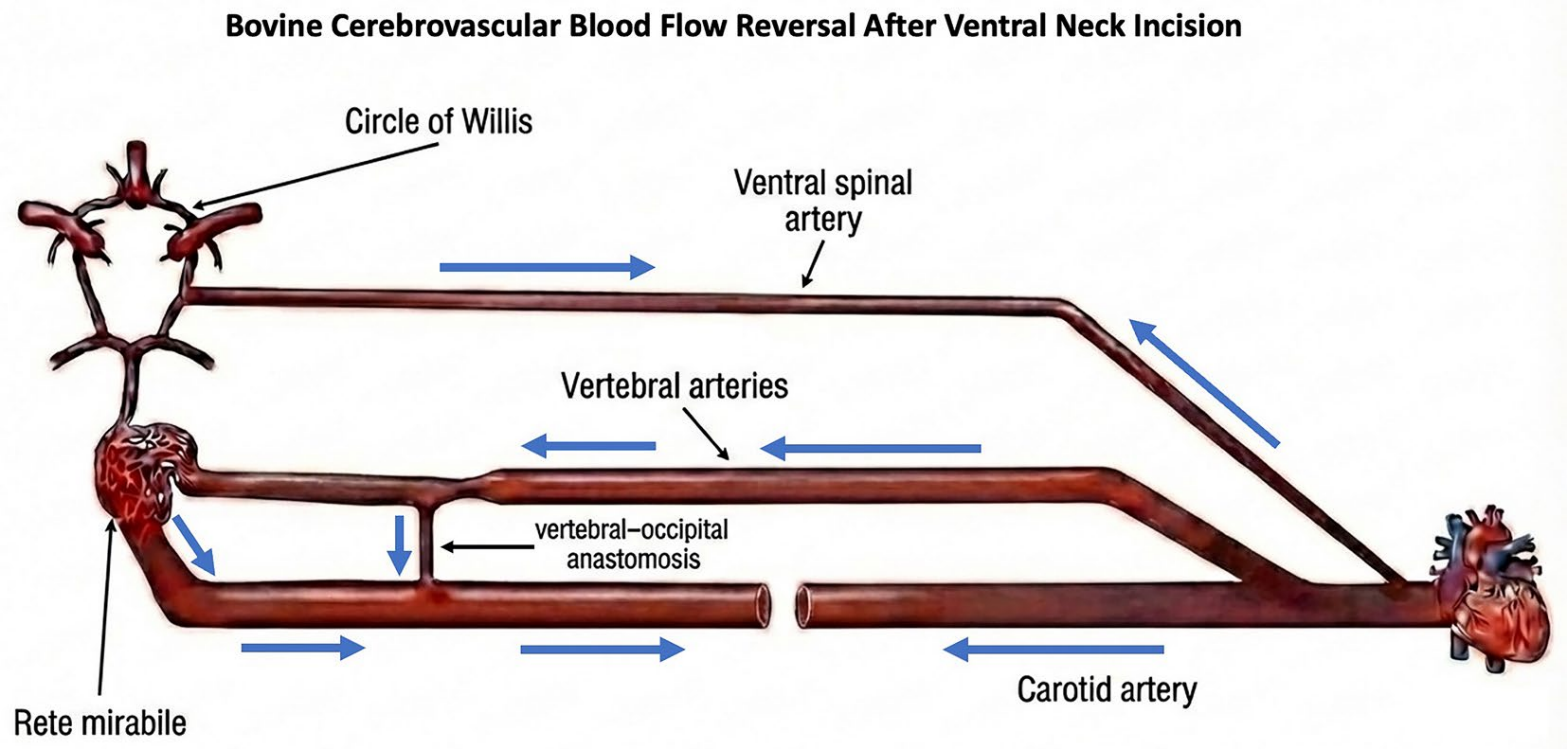
Depiction of the arterial circulation following ventral neck incision without stunning, highlighting the transition from a closed to an open circulatory system. Transection of the carotid arteries results in rapid loss of arterial pressure and blood volume, preventing effective cerebral perfusion despite anatomical continuity of the vertebral arterial pathways. The blue arrows depict the flow reversal following ventral neck incision.

The bovine cerebrovascular system supplies the cerebral cortex from both the ventrally located carotid arteries and the dorsal vertebral arteries (1). At the base of the bovine brain, there is a complex, net-like network of small blood vessels known as the *rete mirabile* which plays a key role in regulating cerebral blood flow. Prior to the *rete mirabile*, the ventral and dorsal systems are connected by the vertebral-occipital anastomosis (2–4). In the intact circulatory system, this anastomosis serves as a conduit for blood flow from the carotid arteries to the vertebral arteries (5) (Figure 1). In the context of slaughter by exsanguination via ventral neck incision (SEVNI), pressure alterations from cut carotids cause blood flow pattern reversal, where blood from the vertebral arteries, by way of the anastomosis, travels into the carotids toward the rostral end of the cut along the path of least resistance (2, 6) (Figure 2).

When animal slaughter is performed for the purpose of food consumption, conventional slaughter with stunning is generally applied on animal welfare grounds, as it is intended to induce rapid unconsciousness and insensibility before bleeding. Stunning can take several forms. For bovines, it is generally done through the physical disruption of brain activity (e.g., penetrating captive bolt, gunshot). In the Jewish religion, shechita is the traditional method of slaughter of mammals and birds designated (‘kosher’) for dietary consumption. A trained worker (*shochet*) uses a surgically sharp knife (*chalaf*) that must be twice as long as the width of the animal’s neck. The blade must be perfectly smooth and free of any nicks (7, 8). The *shochet* cuts the carotid arteries, jugular veins, trachea, esophagus, the *vagus* and sympathetic nerves in a swift, uninterrupted movement. Knives must be checked for sharpness after each shechita incision and before the next (7–10). Similarly, in the Islamic religion, halal slaughter requires specific conditions such as the animal must be healthy, and the slaughter must involve a swift throat incision that severs the windpipe, esophagus, and major blood vessels (8). Neither shechita nor halal permits preslaughter stunning (11).

In the context of SEVNI and for the purpose of this review, LOC in cattle is defined as the loss of cortical awareness, reflecting failure of cerebral cortical function required for conscious experience. Because pain perception requires conscious cortical processing, LOC necessarily entails loss of sensibility and inability to perceive pain. The scientific literature asserts that LOC in bovines after slaughter without stunning, such as in shechita and halal, is a delayed progressive process because brain perfusion in general can be maintained by the unsevered vertebral arteries (12–16). Von Holleben (16) notes, “The vertebral arteries of cattle are not severed by the neck cut due to their passage close to the spinal cord. Unlike sheep, the vertebral arteries in cattle are capable of maintaining the cerebral blood flow”. Similarly, Grandin (13) observes, “In cattle, the vertebral arteries can still supply blood to the brain after the carotids are cut”. Terlouw (14) adds, “…in cattle, the vertebral artery protected by the vertebrae is not cut during bleeding and can continue to irrigate the brain after bleeding”. Finally, Verhoeven (15) quoting Daly (12) explains, “Prolonged consciousness in cattle is believed to be caused by… First, the vertebral artery [which] stays intact in bovine when the neck is cut and continues to supply blood to the brain”. Delayed LOC is problematic as this may subject animals to unnecessary pain, fear and distress, raising significant animal welfare concerns (17). These conclusions require further examination because they are largely derived from aggregation of studies with heterogeneous methodologies, disparate measurement tools, differing experimental endpoints, and inconsistent definitions of LOC, which is occasionally conflated with later markers of brainstem failure or brain death rather than cortical LOC (18).

The vertebral artery hypothesis is largely predicated upon a small number of experiments by Baldwin and Bell (1) and Blackman (19), both of which independently concluded that the vertebral artery can continue to perfuse the bovine brain following slaughter or vessel occlusion. However, the central question is not whether vertebral arteries remain anatomically patent or exhibit residual flow after SEVNI, but whether cerebral perfusion remains above the physiological threshold required to sustain integrated cortical processing and conscious experience. From a cognitive neuroscience perspective, consciousness is a systems-level phenomenon that depends on adequate cortical perfusion and network integration (20), not on vascular continuity alone. In contrast, Levinger (9) and Rosen (21) contend that in cattle, the precipitous decline in cerebral blood flow and pressure following SEVNI renders vertebral artery perfusion insignificant, and therefore LOC is in fact rapid rather than progressive. More recent reviews also suggest that SEVNI leads to rapid LOC, resulting from the immediate and complete cessation of cerebral blood flow (22) and the subsequent loss of cortical function (18). Given the ongoing debate over the vertebral arteries’ role in maintaining cerebral perfusion after slaughter, this review aims to systematically examine the literature to clarify their contribution to cerebral perfusion and determine their functional significance, if any.

## Methods

**Figure 3:**
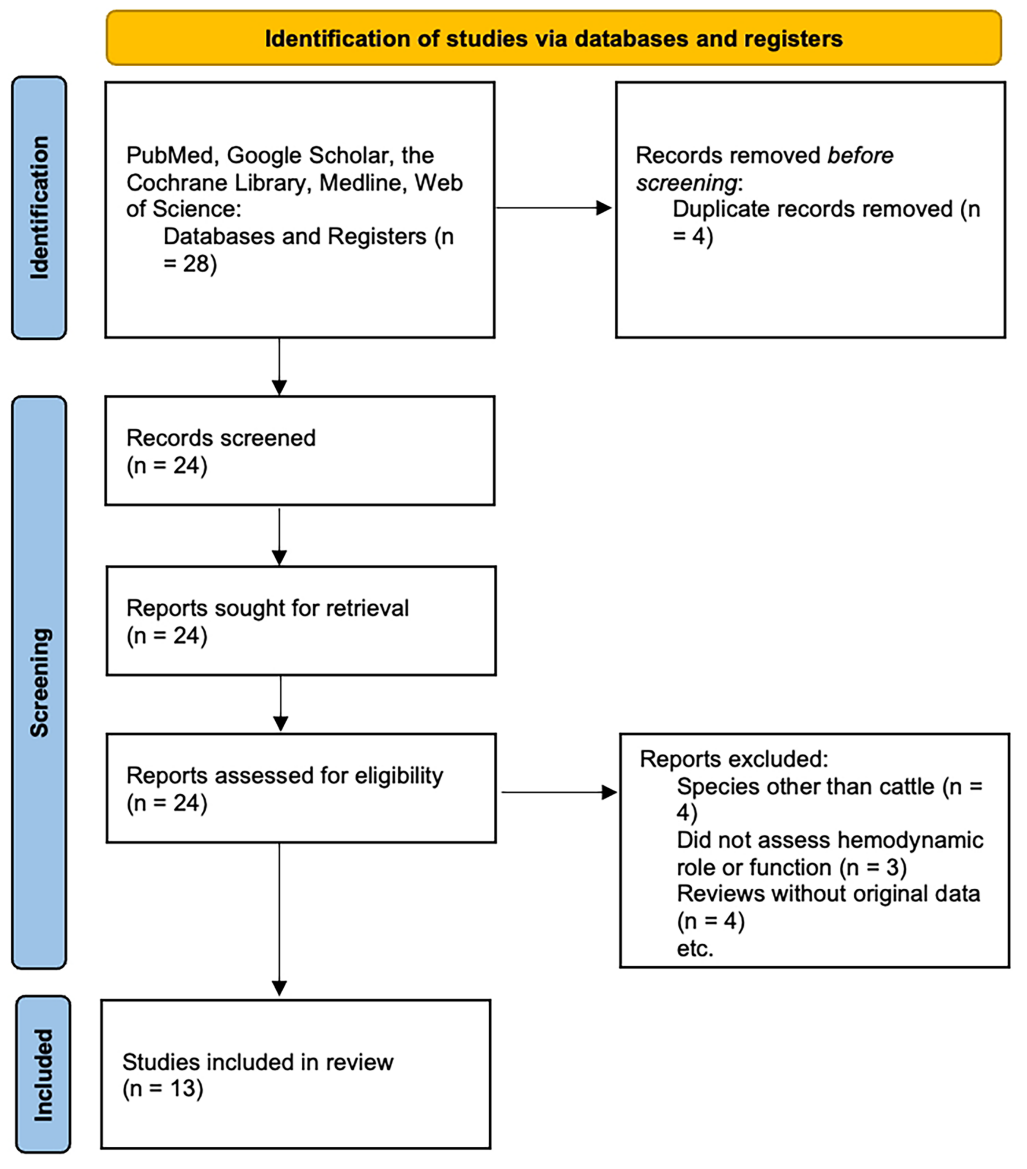
PRISMA flow diagram.

A non-registered systematic review of the literature was conducted to clarify the hemodynamic and functional significance of the vertebral arteries in cattle in the context of SEVNI. PubMed, Google Scholar, the Cochrane Library, Medline, and the Web of Science databases were searched with key words (calf OR bovine) AND vertebral artery AND (blood flow OR pressure) AND slaughter AND (“cerebral perfusion” OR “cerebral circulation” OR “cortical function”); vertebral artery AND (calf OR bovine) AND blood pressure AND (shechita OR halal) AND (“cerebral perfusion” OR “cerebral circulation” OR “cortical function”). The last search date was February 10 2026. The complete database-specific search strategies, including exact search strings, search dates, and applied filters, are provided in Supplementary material 1 to ensure transparency and reproducibility. Eligible study designs included experimental, observational, or physiological studies that reported direct or indirect evaluations or measurements of hemodynamics and the functional significance of the vertebral arteries of cattle. Exclusion criteria were studies in species other than cattle, studies not measuring vertebral artery hemodynamics or not commenting on function, reviews or commentaries without original data and abstracts without full text. Reference lists of all the articles obtained were manually reviewed to identify additional relevant studies. Twenty-eight articles were screened for relevance. Titles and abstracts were screened independently by the co-authors. Full texts of potentially eligible studies were assessed for inclusion. Disagreements at any stage were resolved through discussion and consensus. After screening and full-text review, 13 studies met the inclusion criteria (Figure 3). For each study the authors, year of publication, number of animals included in the study, use of anesthesia, study design, method of slaughter if applicable, and key findings were extracted and tabulated (Table 1). To decrease the risk of bias, the co-authors independently reviewed all the articles. Risk of bias was also assessed using the ROBINS-I framework, a structured tool for evaluating bias in non-randomized studies across multiple methodological domains, adapted for experimental, observational, and physiological studies (23). Each included study was evaluated across standard ROBINS-I domains, including confounding, participant selection, intervention classification, deviations from intended interventions, missing data, outcome measurement, and selective reporting. Data were extracted from studies employing measures of cerebral hemodynamics, cortical electrical activity, and behavioral observation to assess the outcome effect of LOC, as defined above (see Introduction). Given substantial heterogeneity in study design and measurement modalities, quantitative pooling and sensitivity analyses were not methodologically appropriate; therefore, results were synthesized narratively. Certainty in the body of evidence for each outcome domain was assessed qualitatively using a structured, domain-based approach adapted from GRADE principles, considering risk of bias, consistency, directness, biological plausibility, and coherence across studies. A formal GRADE rating was not performed because outcomes were mechanistic and not amenable to quantitative pooling. The results of the studies are summarized (Table 1). PRISMA guidelines for reporting of systematic reviews were followed (24). This study received no external funding.

**Table 1:** Summary of studies investigating vertebral artery circulation and cerebral perfusion in cattle.

Author (Year)	Size (N)	Anesthesia	Study design	Intervention type	Key findings
Hemodynamics studies					
Leiben (25)	1 calf	N/A	Measured blood pressure in aorta and vertebral arteries	Shechita	>50%, drop in vertebral artery pressure within 2–3 s
Sahlstedt (26)	1 cow	N/A	Vertebral blood flow measurement	Shechita	Flow drops to about 1/30th to 1/40th (2.5–3%) of the normal amount within 20 s
Lieben (27)	1 calf	General anesthesia	Vertebral blood flow measurement (contrast dye)	Shechita	Contrast dye injected into the vertebral does not reach brain tissue post shechita
Dukes (5)	6 calves	N/A	Exp 1. Vertebral blood pressure measurement	Shechita	>60% loss of vertebral artery pressure within 2 s
	8 calves	General anesthesia	Exp 2. Vertebral blood flow measurement	N/A	Vertebral flow of 27.7 ml/min Carotid flow 87 to 120 mL/min
				Clamping carotids	Increase in vertebral flow by 345%
				Clamping carotids + anastomosis	No increase in vertebral artery flow
Sporri (6)	1 cow	Local anesthesia	Blood pressure: internal maxillary	Carotid severance	~128 mmHg systolic to 50 mmHg in 1 s; then to 20 mmHg for 25 s
			Femoral		180 mmHg systolic to 80 mmHg in 25 s; then to 28 mmHg at 100 s
			Ulnar		180 mmHg systolic to 80 mmHg in 25 s; then to 28 mmHg at 100 s
Baldwin and Bell (1)	10 calves	General anesthesia	Vertebral artery flow	Clamping carotids	Single carotid clamp showed 107% increase in vertebral blood flow Second carotid clamp “further increase” *
Anil (29)	5 calves	General anesthesia	Vertebral artery flow	Lateral neck stick	Prompt 70% reduction in vertebral flow Sustained 30% persistent flow for 3 min (animal hoisted by hind legs)
Levinger (9)	? calves	General anesthesia	Contrast dye injected into left ventricle	Shechita	Histological examination of brain showed no dye material
Blackman (19)	2 calves	General anesthesia	Contrast dye via intracardiac catheter	Severing both carotid arteries and jugular veins	Presence of dye in detected for 100 s
Bager et al. (2)	12 calves	N/A	Jugular blood flow measurement**	Lateral neck stick	Total cerebral blood flow of 3.6 mL/min/100 g of brain tissue or 4.8% of normal
Electrophysiology studies					
Baldwin and Bell (30)	6 calves	General anesthesia	EEG activity	Clamping carotids	Carotid clamping had no effect on EEG activity
Shaw et al. (31)	4 calves	N/A	EEG loss of activity	Vertebral ligation	43 ± 13 s
				Carotid severance + rostral end ligation	51 ± 25 s No significant difference in time to loss of spontaneous cortical activity on EEG
Behavior observation studies					
Bongert (32)	2 calves	Local anesthesia	Behavior observation	Ligated vertebral arteries	Observed reflex kicking behavior identical to animals with intact vertebral

## Results

Across the included studies, the overall risk of bias was judged to be moderate, reflecting heterogeneity in study design, historical methodological limitations, and unavoidable confounding inherent to experimental and physiological investigations. Risks were lowest for intervention classification and missing data, and higher for confounding, deviations from intended interventions, and selective reporting, particularly in older studies lacking preregistration and standardized reporting. A full ROBINS-I–based risk of bias assessment for all included studies is provided in Supplementary material 2.

### Hemodynamic studies

Lieben et al. (25) used a manometer to measure blood pressure in the vertebral arteries and the aorta of calves before and after shechita. They observed a significant, >50%, drop in vertebral artery pressure within 2–3 s of shechita.

Sahlstedt et al. (26) measured blood flow through the vertebral artery before and after shechita. The study demonstrated that while the vertebral arteries supply approximately 16 to 20% of bovine cerebral flow under normal conditions, this flow drops to about 1/30th to 1/40th (2.5–3%) of the normal amount within 20 s of shechita.

Lieben (27) measured vertebral artery blood flow through the injection of various contrast materials. The authors demonstrated that prior to shechita, contrast injected into the vertebral artery of an anesthetized bull calf reached the brain tissue. If administered as early as 5 s after shechita, the contrast failed to reach the brain.

Dukes et al. (28) measured blood pressure in the vertebral arteries of bovines during shechita and found that pressures dropped, on average, by 50.2 mmHg/s from baseline of 135–160 mmHg during the first 3 s; and by more than 60% within 2 s. In the same paper, Dukes also measured vertebral blood flow of 8 anesthetized calves with functional (non-ligated) carotids. The results showed a flow of 27.7 mL/min in the vertebral arteries, while carotids blood flow was 87 to 120 mL/min. The clamping of carotids in calves induced a modest increase of blood pressure of only 5–10 mm/Hg in vertebral arteries, while blood flow in the vertebral arteries greatly increased by 345%. This increase of blood flow in the vertebral arteries was not observed if both the carotid arteries and the vertebral-occipital anastomoses were clamped.

Sporri (6) measured the blood pressure in the internal maxillary artery (head); ulnar artery and femoral artery (periphery) in cattle before and after cutting of the carotids. In the peripheral ulnar and femoral arteries, blood pressure decreased from 180 mmHg systolic to 80 mmHg in 25 s. Pressure in the internal maxillary artery decreased by 80 mmHg, from 128 mmHg to 50 mmHg, within one second.

Baldwin and Bell (1) measured blood flow in the vertebral arteries under normal conditions and under various conditions of arterial clamping without cutting. Clamping a single carotid artery caused a 107% increase in vertebral blood flow and clamping a second carotid caused a further increase in vertebral flow (1).

Levinger (9) injected India ink into the left ventricle of anesthetized cattle immediately prior to shechita. Histological examination of the brain and other vital organs showed that the liver and the kidneys contained considerable amounts of ink, while only small amounts of dye material were detected on the inferior surface of the brain, and no ink was observed in the cerebral cortex.

Blackman et al. (19) injected methylene blue via an intracardiac catheter into two calves while the cerebral cortex was surgically exposed. After severing both carotid arteries and jugular veins, the authors observed diffusion of dye in the cortex for over 100 s.

Bager, Devine and Gilbert (2) measured jugular blood flow during bleeding after cutting both carotid arteries. The paper assumed that jugular blood flow following carotid cutting represents vertebral flow. The average jugular blood collected after carotid severance was the equivalent of a total cerebral blood flow of 3.6 mL/min/100 g of brain tissue or 4.8% of normal.

Anil et al. (29) measured vertebral blood flow in calves after a neck stick (lateral stab in the neck, followed by a transverse incision in a retrograde way to sever the soft tissues and the skin in the neck). The authors noted a “prompt” reduction in vertebral artery blood flow (<70%) followed by sustained vertebral flow at approximately 30% of its initial level for 3 min.

### Electrophysiology studies

Baldwin and Bell (30) also measured electroencephalogram (EEG) activity in the brain of calves under normal conditions and after bilateral clamping of the carotid arteries (no cutting). They found that bilateral carotid clamping had no effect on the EEG.

Shaw et al. (31) examined the role of the vertebral arteries of calves in determining the time to loss of spontaneous activity of an EEG. In the first set of experiments, the vertebral arteries were ligated. Loss of EEG activity was at 43 ± 13 s. In the second set of experiments, carotid artery severance was rapidly followed by ligation of the rostral end of the cut to ensure vertebral blood passed to the brain. Loss of EEG activity was at 51 ± 25 s. The authors report no significant difference between the two groups.

### Behavior observation

Bongert et al. (32) experimentally ligated the vertebral arteries of cattle prior to shechita. Under these conditions, post shechita, the author observed reflex kicking behavior identical to that seen in animals with intact vertebral circulation.

Overall certainty in the hemodynamic evidence was judged to be moderate to high, supported by consistent findings across multiple experimental studies and strong physiological plausibility. Certainty in electrophysiological evidence was moderate, while behavioral observation provided low to moderate certainty due to the subjective nature of the assessment.

## Discussion

This systematic review evaluated the literature reporting on the role of the vertebral arteries in maintaining cerebral blood flow, pressure, and cortical function following SEVNI (without stunning) in cattle. Importantly, the relevance of vertebral circulation after SEVNI cannot be evaluated solely in terms of residual flow or vessel patency. Across the reviewed studies, determinants of LOC, as defined in this review, were derived from three methodological domains. Hemodynamic studies are based on the physiological principle that cerebral blood flow and perfusion pressure must be maintained above minimum thresholds to support integrated cortical function and conscious awareness; substantial reductions in cerebral perfusion are therefore incompatible with sustained cortical function and imply LOC. Electrophysiological studies infer LOC from loss of organized cortical activity as measured by EEG. Behavioral observation studies infer LOC from the loss of brainstem reflexes or the emergence of motor responses (e.g., kicking reflexes), which typically occur only in states of severe cortical and subcortical dysfunction and thus imply LOC. Across the reviewed studies, the evidence indicates that cerebral perfusion by the vertebral arteries following SEVNI is likely minimal or negligible. This is in contrast to several notable review articles in the animal science literature which assert that brain perfusion after SEVNI can be maintained by the unsevered vertebral arteries (13–16). Importantly, many of these review articles cite Baldwin and Bell (1) and Blackman et al. (19) as their primary sources in support of the role of the vertebral artery in maintaining cerebral perfusion in cattle following SEVNI. Baldwin and Bell (1) reported increased vertebral flow following carotid clamping and no change on calf brain EEG activity (30) suggesting that the vertebral arteries can sustain cortical activity under these conditions. However, it is important to note that carotid clamping preserves a closed (intact) vascular system in which intravascular pressure remains intact and is often increased upstream of the clamp (33), differing fundamentally from SEVNI where the system is breached and pressure collapses due to rapid hemorrhage. In a closed system, pressure may be redirected through collateral channels such as the vertebral occipital anastomosis despite altered flow, but this condition is not representative of the hemodynamic realities of SEVNI. Thus, these findings cannot be reliably extrapolated to support the argument that vertebral circulation sustains cortical perfusion after SEVNI. The clamping model is altogether a different situation.

Blackman et al. (19) did observe methylene blue dye passing through the cerebral hemispheres after carotid and jugular severance. However, this study was purely qualitative and did not indicate how much dye was present. The presence of dye establishes that some flow persisted, but provides no information about perfusion pressure or the adequacy of flow to sustain neuronal activity. Flow without pressure, particularly in the context of catastrophic hemorrhage, cannot maintain cortical perfusion or cortical function. Alternatively, passive diffusion along a concentration gradient, consistent with Fick’s law (which governs the movement of substances from regions of higher to lower concentration in the absence of active flow), may hypothetically explain why dye is seen in the cerebral hemispheres under these conditions (34). Taken together, neither Baldwin and Bell (1) nor Blackman et al. (19) provide convincing evidence to support the argument that cortical function is maintained by vertebral arterial flow after a definitive incision through the carotids.

Verhoeven (15) refers to Daly (12) as evidence that vertebral artery flow can sustain cortical perfusion following SEVNI. Daly et al. (12), in the introduction to their paper entitled *Cortical Function in Cattle During Slaughter: Conventional Captive Bolt Stunning Followed by Exsanguination Compared with Shechita Slaughter*, discusses the role of the vertebral arteries in cerebral perfusion after SEVNI. They cite a study by Dukes (5) in which, Daly claims, Dukes “recorded significant blood pressure in the vertebral arteries after shechita for up to 27 seconds.” The authors present this supporting a role for the vertebral arteries in maintaining cerebral perfusion following SEVNI (5, 12). In fact, Daly is citing the part of Dukes’ paper that deals with blood flow, not blood pressure. When Dukes examines vertebral artery pressure he concludes as follows, contrary to what Daly reports: “It may be assumed, therefore, that in most cases of slaughtering in which the great vessels of the neck are severed, the vertebral arteries contribute little, if any, blood to the cerebral circulation after the cut” (5). Moreover, as noted earlier in the context of Blackman et al. (19), the mere presence of flow provides no information about perfusion pressure or whether the flow is sufficient to sustain cortical function.

Evidence from both behavioral and electrophysiological studies supports the conclusion that vertebral artery perfusion does not sustain cortical activity after SEVNI, while recognizing that EEG, although an objective measure of cortical electrical activity, represents an indirect surrogate for states of consciousness. Bongert (32) observed identical reflexive motor activity in cattle with and without vertebral ligation, supporting the conclusion that post-cut movements are spinally mediated and not dependent on cortical activity/function or vertebral blood flow. Shaw et al. (31) directly assessed cortical activity with EEG and found no difference in time to EEG silence whether vertebral arteries were ligated or left intact, demonstrating that vertebral circulation had no meaningful role in prolonging cortical function after SEVNI.

Several experimental studies provide direct quantitative evidence of the rapid collapse of vertebral and cerebral arterial pressures immediately following SEVNI. Lieben (25, 27) demonstrated both a greater than 50% drop in vertebral arterial pressure within seconds of shechita and the failure of injected contrast dye to reach the brain if administered just five seconds post-incision, providing compelling evidence of the near-immediate cessation of vertebral cortical perfusion. Dukes (28) documented precipitous declines in vertebral artery pressure, with more than 60% reduction within two seconds, falling below levels required to sustain consciousness. Spörri (6) showed that pressure in the internal maxillary artery, a proxy for cerebral perfusion, dropped by >80 mmHg within one second after carotid severance, further demonstrating the impossibility of sustained cortical function. Within one second of carotid severance during shechita, blood pressure in all arteries supplying the brain, including the common carotid, distal vertebral, and internal maxillary arteries, falls to approximately one-fifth of the initial value (20–30 mmHg) (6).

Further hemodynamic studies quantify the rapid and profound decline in vertebral and cerebral blood flow following SEVNI, providing additional evidence that cortical function cannot be sustained. Sahlstedt (26) found that vertebral flow falls to less than 3% of baseline within 20 s, a level incompatible with maintaining consciousness. Levinger (9) found only trace amounts of contrast dye in the inferior brain and none in the cortex after shechita, while Bager et al. (2) reported jugular venous outflow equivalent to only 4.8% of normal cerebral flow, confirming marked hypoperfusion. Anil et al. (29) measured a sustained 30% residual vertebral flow for several minutes post-slaughter. Since the vertebral arteries contribute only 20% of total bovine cerebral flow under normal conditions (26), a 70% reduction leaves only ~6% of total cerebral perfusion, an amount that is insufficient to sustain cortical function. Furthermore, a 70% “prompt” (authors’ words) reduction in vertebral flow alone would be sufficient to abolish consciousness. Reductions of 30–50% in cerebral blood flow or pressure are known to cause LOC in humans (35–37). Although species differences in cerebrovascular anatomy and metabolic demand must be acknowledged, human data provide a useful physiological reference, supporting the inference that a comparably rapid and severe reduction in cerebral perfusion in cattle would be expected to result in LOC (18, 22). Additionally, in the Anil study some calves were shackled and hoisted by their hind legs after the cut, a factor that may have influenced flow in the vertebral arteries (29).

These findings have direct relevance for animal welfare assessment during slaughter. Accurate determination of LOC is central to evaluating insensibility, and such assessments should be aligned with the underlying neurophysiological mechanism of unconsciousness. When LOC following SEVNI results from rapid cortical hypoperfusion, indicators that primarily reflect delayed brainstem failure such as the corneal or palpebral reflexes may be physiologically misaligned with the true endpoint of interest. From an animal welfare perspective, this supports prioritizing cortically mediated indicators, such as loss of the menace reflex, which depend on intact cortical processing and therefore more directly reflect cortical LOC.

This review is limited by heterogeneity in study designs, outcome measures, and experimental techniques, particularly in the older literature where methodological constraints may have reduced measurement precision. This heterogeneity limits interpretability and comparability across studies. The available literature meeting predefined inclusion criteria was limited, resulting in a relatively small number of studies eligible for detailed analysis; this reflects the scarcity of experimental work in this area rather than selective exclusion. Publication bias cannot be entirely ruled out, although both supportive and non-supportive findings were included. These factors precluded quantitative pooling through meta-analysis, so the conclusions presented here are based on a narrative synthesis of the available evidence. The review was not preregistered, which is acknowledged as a limitation; however, preregistration was not undertaken because the review did not address a single predefined quantitative outcome amenable to prospective registration. Future studies employing modern hemodynamic and neurophysiological techniques, when possible, should be prioritized over additional animal experimentation, which carries a risk of unnecessary harm and pain and should therefore be avoided in the interest of animal welfare.

In conclusion, this literature review did not find evidence indicating that the vertebral arteries can deliver sufficient blood flow or pressure to the cerebral cortex after SEVNI to sustain cortical function. The available experimental, physiological, and functional data demonstrates that vertebral perfusion likely falls rapidly to negligible levels, insufficient to maintain cortical function. In practical terms, this suggests that in real world slaughterhouse conditions, concerns that vertebral perfusion might prolong consciousness after the neck cut is unlikely based on the available evidence, supporting the efficacy of properly performed SEVNI in inducing rapid LOC and therefore insensibility.

## Data Availability

The original contributions presented in the study are included in the article/Supplementary material, further inquiries can be directed to the corresponding author.
