# Supplementary material for: Vertebral artery contribution to cerebral cortex perfusion in cattle after slaughter by ventral neck incision: a systematic review
Source: Front Vet Sci. 2026 Feb 25;13:1760260. doi: 10.3389/fvets.2026.1760260 (PMC12977232; doi:10.3389/fvets.2026.1760260)
Supplement: Supplementary file 1 [file Supplementary_file_1.docx]

**Supplementary Material S1: Detailed Search Strategy**
 **Databases and Search Dates**
A comprehensive literature search was conducted to identify relevant studies published from the earliest records available in each database, including early 20th-century publications, through February 10, 2026. The following electronic databases were searched: PubMed (MEDLINE), Web of Science Core Collection, Cochrane Library, Medline, and Google Scholar. All searches were last updated on February 10, 2026.

Given the historical nature of foundational physiological studies in this field, no lower date limit was applied in any database in order to capture early experimental and anatomical work, including studies published in the 1920s and earlier.

**Search Strategy and Terms**
Search strategies were tailored to the indexing structure and search syntax of each database. No language restrictions were applied at the search stage. Where necessary, older terminology and historical nomenclature were accounted for through iterative searching and manual reference screening.

*PubMed (MEDLINE):*
(calf OR bovine OR cattle) AND "vertebral artery" AND ("blood flow" OR "blood pressure" OR perfusion) AND slaughter AND ("cerebral perfusion" OR "cerebral circulation" OR cortex OR consciousness)

*Religious slaughter–specific PubMed search:*
"vertebral artery" AND (bovine OR cattle OR calf) AND ("blood pressure" OR perfusion) AND (shechita OR shechitah OR halal OR dhabihah) AND ("cerebral perfusion" OR "cerebral circulation" OR cortical)

*Web of Science Core Collection:*
TS=(bovine OR cattle OR calf) AND TS=("vertebral artery") AND TS=("blood flow" OR "blood pressure" OR perfusion) AND TS=(slaughter OR bleeding OR shechita OR halal)

*MEDLINE (alternative interfaces):*
bovine AND vertebral artery AND cerebral circulation

*Cochrane Library:*
(bovine OR cattle) AND "vertebral artery" AND ("cerebral perfusion" OR "cerebral circulation")

*Google Scholar:*Due to limited Boolean functionality, iterative searches were performed using combinations of terms including:
bovine vertebral artery cerebral perfusion
cattle vertebral artery cerebral blood flow
bovine cerebral circulation slaughter
vertebral artery cattle bleeding

For each query, the first 200 results sorted by relevance were screened. Additional historical studies were identified through citation chaining.

**Filters and Limits**
Species: Cattle (Bos taurus)
Study types: Experimental, observational, anatomical, physiological
Exclusions: Reviews without original data, editorials, opinion pieces, conference abstracts without full text
Time limits: None applied

**Study Selection and Data Extraction**Titles and abstracts were screened independently by co-authors. Full texts of potentially eligible studies were assessed for inclusion. Disagreements were resolved by consensus. Reference lists of included studies were manually reviewed to identify additional relevant publications.

Data extracted included authors, year of publication, study design, slaughter or experimental method, hemodynamic measures, indicators of cerebral perfusion or function, and key findings.

**Synthesis**Given substantial heterogeneity in study designs, methods, outcome measures, and historical context, results were synthesized narratively. Quantitative pooling and meta-analysis were not performed. Reporting followed PRISMA guidelines where applicable.
