# Supplementary material for: Vertebral artery contribution to cerebral cortex perfusion in cattle after slaughter by ventral neck incision: a systematic review
Source: Front Vet Sci. 2026 Feb 25;13:1760260. doi: 10.3389/fvets.2026.1760260 (PMC12977232; doi:10.3389/fvets.2026.1760260)
Supplement: Supplementary file 2 [file Supplementary_file_2.docx]

**Risk of Bias Assessment (ROBINS-I Framework)**

Risk of bias in the included studies was assessed using the ROBINS-I (Risk Of Bias In Non-randomized Studies of Interventions) framework, adapted for experimental, observational, and physiological studies. Each study was evaluated across the seven ROBINS-I bias domains. Given the historical nature and heterogeneity of the literature, assessments were qualitative and domain-based rather than numerical.

**1. Bias Due to Confounding**

*Moderate risk of bias*
Several studies were subject to important confounders, including use of anesthesia, carotid clamping rather than carotid sectioning, variations in animal positioning (e.g., hoisting), and differences in timing of post-incision measurements. These factors may influence cerebrovascular pressure and flow. However, the primary direction of effect across studies was consistent despite these confounders, reducing the likelihood that confounding alone accounts for the observed findings.

**2. Bias in Selection of Participants into the Study**

*Low to moderate risk of bias*
All participants were cattle. Cattle were typically selected based on availability rather than random sampling, and allocation procedures were rarely described. However, as outcomes were physiological rather than comparative between intervention groups, selection bias is unlikely to have materially affected the internal validity of the reported hemodynamic or electrophysiological measurements.

**3. Bias in Classification of Interventions**

*Low risk of bias.*
Interventions (e.g., shechita, halal, carotid sectioning, carotid clamping, vertebral ligation) were generally well defined and clearly distinguishable. Misclassification of the intervention status was unlikely, particularly in studies involving direct surgical manipulation or clearly specified slaughter techniques.

**4. Bias Due to Deviations from Intended Interventions**

*Moderate risk of bias*
Some studies deviated from conditions representative of commercial slaughter, including the use of vascular clamping instead of sectioning and experimental manipulations performed in anesthetized animals. These deviations limit direct extrapolation to real-world SEVNI but do not invalidate mechanistic conclusions regarding pressure collapse and flow redistribution in an open vascular system.

**5. Bias Due to Missing Data**

*Low risk of bias*
Most studies reported complete outcome data for the physiological variables measured. Attrition and missing data were uncommon and, when present, were not clearly associated with outcome direction.

**6. Bias in Measurement of Outcomes**

*Low to moderate risk of bias*
Studies using direct quantitative measures (arterial pressure, blood flow, EEG activity) were judged at low risk of measurement bias. In contrast, studies relying on qualitative indicators (e.g., visual assessment of dye distribution or behavioral observation) were considered at moderate risk due to potential observer subjectivity and lack of standardized thresholds. Blinding of outcome assessors was generally not reported.

**7. Bias in Selection of the Reported Result**

*Moderate risk of bia*s
Given the absence of preregistered protocols, selective reporting cannot be excluded. Nonetheless, the inclusion of studies with both supportive and non-supportive findings regarding vertebral artery contribution reduces concern for systematic reporting bias favoring a single conclusion.

**Overall Risk of Bias Judgment**

Overall, the body of evidence was judged to have **moderate** risk of bias. This assessment reflects heterogeneity in study design, methodological limitations, and unavoidable confounding inherent to physiological experimentation. Importantly, no single study at critical risk of bias dominated the evidence base, and conclusions were supported by convergent findings across independent anatomical, hemodynamic, electrophysiological and behavioral approaches, strengthening confidence in the overall interpretation.
